# Increased Frequency of Aneuploidy in Long-Lived Spermatozoa

**DOI:** 10.1371/journal.pone.0114600

**Published:** 2014-12-09

**Authors:** Young-Ah You, Yoo-Jin Park, Woo-Sung Kwon, Sung-Jae Yoon, Buom-Yong Ryu, Young Ju Kim, Myung-Geol Pang

**Affiliations:** 1 Department of Animal Science and Technology, Chung-Ang University, Anseong, Gyeonggi-Do 456–756, Republic of Korea; 2 Department of Obstetrics and Gynecology, Ewha Womans University, Seoul 158–710, Republic of Korea; St. Georges University of London, United Kingdom

## Abstract

Aneuploidy commonly causes spontaneous abortions, stillbirths, and aneuploid births in humans. Notably, the majority of sex chromosome aneuploidies in live births have a paternal origin. An increased frequency of aneuploidy is also associated with male infertility. However, the dynamics and behavior of aneuploid spermatozoa during fertilization in humans have not been studied in detail. Therefore, we compared the frequency of aneuploidy and euploidy in live spermatozoa from normozoospermic men over a 3-day period. To assess the dynamics and behavior of aneuploid spermatozoa, we simultaneously evaluated sperm viability using the hypo-osmotic swelling test and sperm aneuploidy using fluorescence in situ hybridization. Whereas the frequency of viable euploid spermatozoa significantly decreased over 3 days, the frequency of viable spermatozoa with aneuploidy interestingly showed a time-dependent increase. In addition, spermatozoa with abnormal sex chromosomes survived longer. To compared with spermatozoa with other swelling patterns, those with tail-tip swelling patterns had a lower frequency of aneuploidy at all time points. This study revealed the novel finding that the frequency of aneuploid spermatozoa with fertilization capability significantly increased compared to that of euploid spermatozoa over 3 days, suggesting that aneuploid spermatozoa can survive longer than euploid spermatozoa and have a greater chance of fertilizing oocytes.

## Introduction

Aneuploidy, the presence of extra or missing chromosomes, is a numerical chromosomal abnormality. It contributes to spontaneous abortions, stillbirths, and aneuploid births with severe congenital defects in humans [1], [2]. Aneuploid zygotes that survive beyond implantation are either sex chromosome aneuploids or one of three types of autosomal trisomies (chromosomes 13, 18, and 21) [3]. These aneuploidies arise as de novo mutations or structural rearrangements either during the meiosis of oogenesis or spermatogenesis or during mitosis after fertilization [4]. Assisted reproductive technology data suggest that disomy occurs in 1–17% of spermatozoa in men with normal somatic karyotypes [5], [6] and in 20% of oocytes [7]. Surprisingly, 25% or more of the gametes involved in assisted reproductive technology-derived pregnancies are aneuploid [8]. Most autosomal aneuploidy is maternal in origin. Nevertheless, approximately 80% of aneuploidies associated with sex chromosomes are paternal in origin: 6% of 47, XXX; 50% of 47, XXY; 80% of 45, X; and 100% of 47, XYY cases [9]–[11]. Aneuploidies of the sex chromosomes are present in the general population with a frequency of approximately 1 in 1000 for each syndrome [12].

Spermatozoa are the motile vehicles that transmit the paternal half of the genetic material for fertilization. All men-even those who are healthy and fertile–have some aneuploid spermatozoa [13]. However, an increased frequency of sperm aneuploidy is associated with infertility. A significant increase in the frequency of autosomal and sex chromosome disomy is found in infertile men compared with normal controls [14], [15]. The embryos formed from these aneuploid spermatozoa tend to have a demonstrable background level of aneuploidy and chromosome breakage [16], which can cause overall reproductive failure and increase the incidence of spontaneous abortion and abnormalities at birth [17].

Although the main factors causing sperm aneuploidy are genetically controlled, environmental factors, both physical and chemical, may also be factors in aneuploidy. To date, many studies have investigated increases in the frequency of sperm aneuploidy associated with various environmental factors such as advanced age [18], pollution [19], cigarette smoking, alcohol consumption [20], and cancer chemotherapy [21]. Despite the numerous studies undertaken in this research area, no studies on viability related to sperm aneuploidy have been reported.

This study aimed to determine aneuploidy frequency and its relationship to viability in spermatozoa cultured up to 3 days. Little knowledge about these phenomena is available. Determining whether a relationship exists between sperm viability and nondisjunction requires simultaneous study of both viability and chromosome composition in individual spermatozoa. Spermatozoa were cultured for 3 days, and viability status and chromosomal constitution were evaluated simultaneously in each spermatozoon.

## Materials and Methods

### Semen preparation and sperm culture

All participants were fully informed about the study and did provide written informed consent. Ethical approval was obtained from the Institutional Review Board for Human Research at Chung-Ang University Hospital in Seoul, Korea. Semen samples were obtained via masturbation from 12 normozoospermic donors after 3 days of sexual abstinence. The characteristics (mean ± SEM) of donor semen samples were as follows: volume 2.35±0.23 ml, concentration 94.54±11.62×10^6^/ml, motility 72.57±5.08%, and normal morphology 16.23±1.26%. The presence of non-viable cells and white blood cells was lower than 1×10^6^/ml. The samples were divided into 3 groups randomly, each containing the semen of 4 individual. Sperm count and motility were evaluated according to the World Health Organization semen analysis criteria using a computer-assisted semen analyzer (CTS-60/200; Motion Analysis, Santa Rosa, CA). To obtain an appropriate quantity of semen and eliminate bias in sampling, semen samples from each group were pooled for each replicate. Each pooled sample was diluted with Hepes-buffered modified Tyrode’s albumin-lactate-pyruvate (mTALP) solution containing 0.3% bovine serum albumin (w/v) and centrifuged at 400× *g* for 5 min. The supernatant was carefully discarded, and the pellet was washed twice with 5 ml of mTALP solution. The final sperm concentration was adjusted to 1×10^6^/ml and cultured to maintain motility and viability at 37C in air for 3 days [22].

### Hypo-osmotic swelling (HOS) test

HOS test and pretreatment of spermatozoa were performed using initial mixed samples after liquefaction (day 0) and on day 3. Sperm suspensions (5 mL) was centrifuged (400× *g*, 5 min), and the pellets were resuspended in 5 ml of 37C hypo-osmotic solutions (distilled water: 0.9% NaCl [1∶1], 150 mOsm/kg), mixed well, and incubated at 37C in a water bath for 30 min [23]. After the incubation, the spermatozoa and hypo-osmotic mixture was centrifuged, and the pellets were resuspended in hypo-osmotic solution. A drop of the suspension was smeared onto a clean slide, allowed to air dry, and fixed with fresh fixative (3∶1 mixture of methanol: glacial acetic acid).

### Fluorescence in situ hybridization (FISH)

After decondensation of spermatozoa using 2 mM dithiothreitol/PBS, multicolor FISH using directly labeled DNA probes specific to chromosomes 18, X, and Y (Kreatech, Amsterdam, Netherlands) was performed on the slides previously prepared with spermatozoa for HOS test [24]. To investigate both the autosomes and the sex chromosomes, we performed simultaneous three-color FISH using probe sets for chromosomes X, Y, and 18.

Hybridization mixes were added to prewarmed slides (42C), covered with 22×22 mm coverslips, and sealed with rubber cement. The slides were denatured at 80C for 5 min. All slides were hybridized in a moist chamber for 16 h at 42C. The post-hybridization coverslips were separated from the slides in 50% formamide/2× SSC, and washes were performed at 37C with 50% formamide/2×SSC, pH 7.0, three times followed by 5 minutes with 0.1% NT40/2× SSC, pH 7.0. The slides were rinsed in phosphate-buffered saline for 5 min at room temperature. Coverslips were added over 10 µl antifade solution. The slides were read with a Nikon Microphot-FXA under epifluorescence illumination using ultraviolet BP 340–380/LP 425 and BP 450–490/LP 515 exitation/suppression filters.

### Scoring criteria

**Figure 1:**
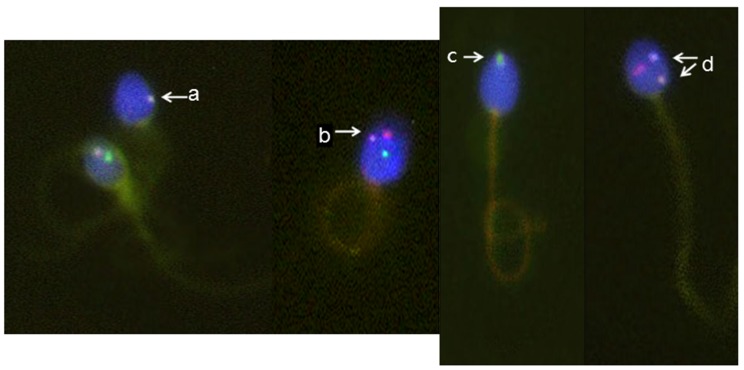
Aneuploid spermatozoa with different tail swelling patterns. Micrographs of live aneuploid spermatozoa showing the swelling patterns. The green fluorescence signal is the Y chromosome, red is X, and orange is chromosome 18: a, live spermatozoon with chromosomes 18; b, live spermatozoon with 18XY; c, live spermatozoon with Y; d, dead spermatozoon 1818X.

We characterized the sperm using the scoring criteria described in our previous study [24]. Briefly, a spermatozoon was scored as disomic for a particular chromosome if it showed two signals for that chromosome and one each for the simultaneously probed chromosomes. If the chromosome was counted as disomic, the distance between the two signals had to equal or be greater than the diameter of one fluorescent domain to distinguish a true aneuploid signal from DNA breakage. In the study, we scored only non-problematic nullisomy. For example, a spermatozoon was scored as nullisomy 18 if it show no signal for chromosome 18 and one each of the simultaneously probed sex chromosome. Therefore we believe problematic nullisomy has been excluded in the study. The various swelling patterns and chromosome abnormalities in each spermatozoon were observed simultaneously under a fluorescence microscope (Fig. 1).

### Statistical analysis

The time-dependent viability of euploid and aneuploid spermatozoa for 3 days was compared using one-way analysis of variance implemented in SPSS (v. 12.0; Chicago, IL, USA). We also analyzed the frequency and type of aneuploidy in relation to sperm tail swelling pattern at each time point. Significance (*p*<0.05) was analyzed with Tukey’s test to determine whether specific groups were significantly different from each another. The data are expressed as the mean frequency of spermatozoa ± standard error of the mean (SEM).

## Results

In total, 24,176 spermatozoa were scored for aneuploidy and tail swelling pattern. The hybridization efficiency was 98–99% for the study. The total number of spermatozoa and the number of live spermatozoa were counted, and the frequency of aneuploidy at each time point was determined (Table 1). We pooled the frequency of aneuploidy for groups of related tail swelling patterns to examine the general relationship between viability and aneuploidy in human spermatozoa.

**Table 1:** Frequency of aneuploidy in total and live spermatozoa from normozoospermic men.

	Day 0	Day 3
No. of sperm	5172	9435
No. of live sperm	3336	3344
Sperm viability (%)	64.50±0.94^a^	35.44±2.09^b^
Total aneuploidy (%)	0.66±0.16	0.81±0.28
Aneuploidy in live sperm (%)	0.33±0.04^a^	0.71±0.18^b^

Statistical significance was analyzed using t-test.

The data were expressed as the mean frequency of spermatozoa ± SEM.

a–b indicate a significant difference between Day 0, and Day 3 group (p<0.05).

**Figure 2:**
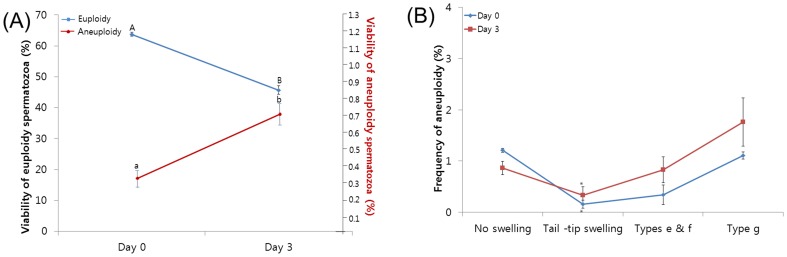
Changes in viability status associated with chromosomal constitution over a 3-day observation period. (**A**) Frequencies of live spermatozoa (blue line) and live aneuploidy (red line). Statistical significance was analyzed using analysis of variance. The data were expressed as the mean frequency of spermatozoa ± SEM. ^A–B^Viability of euploid spermatozoa and aneuploidy spermatozoa (^a–b^) denote significant differences in live frequencies at the indicated time point (*p*<0.05). (**B**) Frequency of aneuploidy related to tail swelling pattern. The data are expressed as the mean frequency of spermatozoa ± SEM. ^*^Spermatozoa with the tail-tip swelling pattern displayed a significantly lower frequency of aneuploidy compared with that in spermatozoa with other patterns at the indicated time point (*p*<0.05).

The frequency of total aneuploidy was the same on days 0 and 3. By day 3 of the culture, the frequency of live spermatozoa time-dependently decreased (blue line, *p*<0.05; see Fig. 2A) while the frequency of live aneuploid spermatozoa had significantly increased (red line, *p*<0.05; see Fig. 2A) compared with values at day 0. The frequency of aneuploidy in live spermatozoa increased significantly over the 3-day period (see Fig. 2A).

The frequency of aneuploidy was related to tail swelling pattern over the 3-day period. Interestingly, spermatozoa with the tail-tip pattern had a frequency of aneuploidy lower than that in spermatozoa with other swelling patterns at all indicated time points (*p*<0.05; see Fig. 2B).

**Figure 3:**
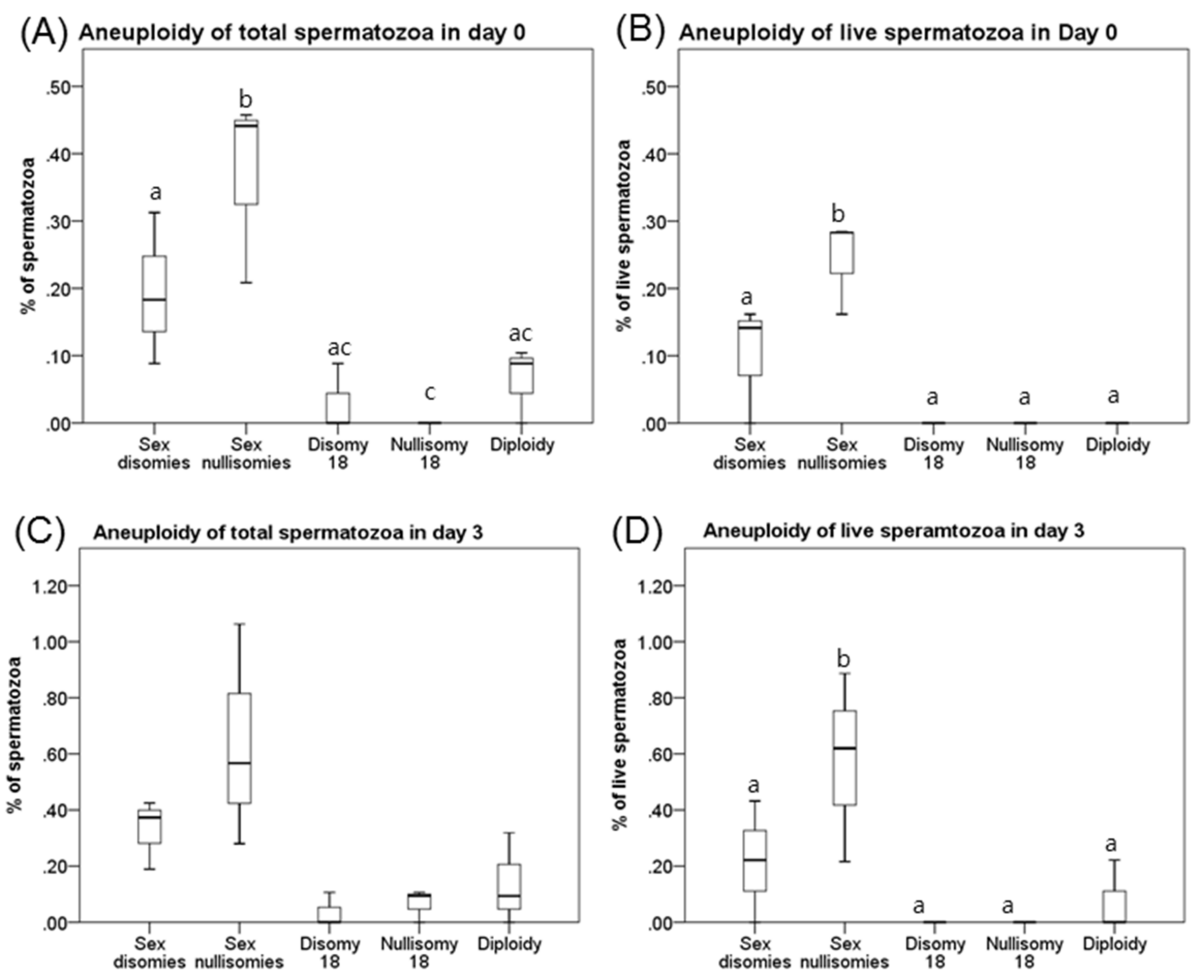
Box plots for the frequency distributions of aneuploidy types in total and live spermatozoa. Aneuploidy frequency of total (**A**) and live (**C**) spermatozoa at day 0. Aneuploidy frequency of total (**B**) and live (**D**) spermatozoa at day 3. ^a–c^Total and live spermatozoa representing significant differences in frequency between aneuploid types at the indicated time point (*p*<0.05).

The frequency of the five types of aneuploidy (sex disomy; sex chromosome nullisomy, disomy, and nullisomy for chromosome 18; and diploidy) differed at each time point in the total and live spermatozoa (*p*<0.05). Spermatozoa with sex chromosome aneuploidy were more prevalent than sperm with chromosome 18 aneuploidy and diploidy. In addition, live diploidy was more frequent on day 3 than on day 0 (*p*<0.05). However, no difference between the distributions of aneuploids in the total sample and live subset was observed at any time point. While the frequency of each types of aneuploidy more significantly increased at Day 3 than Day 0 in live spermatozoa, the frequency of any aneuploidy do not changed between Day 0 and 3 in total spermatozoa (see Fig. 3).

## Discussion

In the present study, we evaluated sperm viability in relation to chromosomal constitution up to 3 days after ejaculation. We simultaneously conducted the HOS test to evaluate functional viability [24] and FISH to determine chromosomal constitution in individual spermatozoa from normozoospermic men [23]. Our findings provide the first vital evidence that the frequency of aneuploidy in live spermatozoa increases significantly over a 3-day period, meaning that aneuploidy is more frequent in long-lived spermatozoa than in fresh, live spermatozoa after ejaculation. Therefore, our results suggest that long-lived aneuploid spermatozoa should have considerable opportunity to fertilize oocytes.

The HOS test evaluates the structural integrity of the membranes of live spermatozoa and allows selection of potentially fertile spermatozoa [25]. The hypo-osmotic swelling test is actually used to discriminate viable from non-viable spermatozoa for intracytoplasmic sperm injection (ICSI) in cases of immotile spermatozoa [26]–[28]. Therefore, we could detect functionally active spermatozoa in both the aneuploidy and the euploidy sperm groups.

All men, even those who are healthy and fertile, have some aneuploid spermatozoa [13]. Human sperm aneuploidy has determined the chromosomal abnormality in nomal semen parameter using the karyotype analysis of human sperm/hamster and human sperm/mouse-oocyte fusion assay [29], [30] or analysis of interphase sperm nuclei using FISH assay [13], [14], [20]. Recently, Templado et al. [31] reviewed the frequency of aneuploidy in spermatozoa from FISH series of healthy donors with a total of 388 donors aged 18–80 years. While the mean disomy frequency was approximately 0.1% for individual autosomes, particularly disomy frequencies for chromosome 21 (0.17%) and the sex chromosomes (0.27%) were increased with more than 2 fold compared with the other autosomes [6]. This increased frequency of sex chromosome aneuploidy was consisted with our results on initial and 3 days in nomozoospermic samples.

In general, sperm aneuploidy is associated with male factor infertility and poor semen quality [32]–[34]. The increased frequency of aneuploidy is negatively correlated with sperm concentration, motility, and morphology in ejaculates. Furthermore, the increased incidence of aneuploidy includes approximately 2% of all men with fertility problems [35], 5% of oligozoospermic men, and 14% of azoospermic men [36]. Pang *et* *al.* [15] have indicated that if the aneuploid frequency of each chromosome shows this slight increase, it is possible to speculate that the total rate of aneuploidy-considering 22 autosomes and two gonosomes-is approximately 33–74% in cases of spermatozoa from oligoasthenoteratozoo-spermic patients. Furthermore, various acquired factors such as advanced age [18], environmental pollution [19], cigarette smoking, alcohol consumption [20], and cancer chemotherapy [21] are closely related to the increased frequency of aneuploidy in spermatozoa.

Taken together, these findings suggest that an increase in aneuploid spermatozoa could pose an increased risk of genetic abnormalities to embryos and offspring. An interesting study of couples with increased aneuploid spermatozoa detected a significant decrease in the number of normal embryos and a significant increase in mosaic embryos [37]–[39]. Fortunately, earlier and stronger selection against most autosomal aneuploid embryos occur in utero (probability of survival to birth of 0–22%). However, most embryos exhibiting sex chromosome aneuploidies cross the pregnancy-birth barrier and have a probability of survival to birth of 55–100% [40]. Collectively, these findings showed that aneuploid spermatozoa deliver their abnormal genetic information from paternal origin for fertilization. Moreover, even without information about how many early embryos develop from aneuploid spermatozoa and expire *in* *vivo*, it is clear that aneuploidy can cause overall reproductive failure through increased incidence of spontaneous abortion and abnormal offspring [16]. In addition, this low rate of successful pregnancy and high rate of implantation failure may be closely associated with aneuploidy in male gametes.

In present study, we analyzed the frequency of aneuploid spermatozoa in relation to their tail swelling patterns to determine the relationship between viability and aneuploidy levels in human spermatozoa. The aneuploidy frequencies in spermatozoa with tail-tip patterns decreased more than those in spermatozoa with other swelling types, including dead spermatozoa with pattern a, at each time point. This result is consistent with our previous finding [23] that the frequency of aneuploidy is decreased in spermatozoa with a tail-tip swelling pattern. This characteristic could be an effective tool for identifying and eliminating aneuploid spermatozoa in *in* *vitro* fertilization programs that incorporate intracytoplasmic sperm injections in men with fertility problems-i.e., immobile spermatozoa, low sperm count.

To the best of our knowledge, this study provided the first definitive evidence of a relationship between viability and aneuploidy in human spermatozoa. Our study revealed that the frequency of aneuploidy in live spermatozoa was significantly increased over a 3-day period. Notably, spermatozoa with abnormal sex chromosomes can be longer-lived than spermatozoa with all other types of aneuploidy. Because long-lived aneuploid spermatozoa could have a greater chance of fertilizing oocytes, they may suppose an increased risk of transmitting sex chromosome abnormalities of paternal origin to offspring.

## References

[1] Hassold T, Abruzzo M, Adkins K, Griffin D, Merrill M, et al (1996) Human aneuploidy: incidence, origin, and etiology. Environ Mol Mutagen 28:167–175. 8908177 10.1002/(SICI)1098-2280(1996)28:3<167::AID-EM2>3.0.CO;2-B

[2] Wyrobek AJ, Marchetti F, Sloter E, Bishop J (2000) Chromosomally defective sperm and their developmental consequences. In: Anderson D, Kaeakaya AE, Sram RJ, eds. Human monitoring after environmental and occupational exposure to chemical and physical agents. Amsterdam: Press IOS, NATO Science series: Series A, Life Sciences. 313:134–150.

[3] Hassold T, Hunt P (2001) To err (meiotically) is human: the genesis of human aneuploidy. Nat Rev Genet 2:280–291. 11283700 10.1038/35066065

[4] Olson SB, Margenis RE (1988) Preferential paternal origin of de novo structural chromosome rearrangements. In: Daniel A. ed. The cytogenetics of mammalian autosomal rearrangements. New York: Press Liss. 583.

[5] Abruzzo MA, Hassold TJ (1995) Etiology of nondisjunction in humans. Environ Mol Mutagen 25:38–47. 7789361 10.1002/em.2850250608

[6] Shi Q, Martin RH (2000) Spontaneous frequencies of aneuploid and diploid sperm in 10 normal Chinese men: assessed by multicolor fluorescence in situ hybridization. Cytogenet Cell Genet 90:79–83. 11060453 10.1159/000015668

[7] Wramsby H, Fredga K, Liedholm P (1987) Chromosome analysis of human oocytes recovered from preovulatory follicles in stimulated cycles. N Engl J Med 316:121–124. 3796682 10.1056/NEJM198701153160301

[8] Vera M, Peinado V, Al-Asmar N, Gruhn J, Rodrigo L, et al. (2012) Human Males Meiosis and Sperm Aneuploidies. In Storchova Z. ed. Aneuploidy in Health and Disease. Croatia; Press InTech, 141–162.

[9] Hoegerman SF, Pang MG, Kearns WG (1995) Sex chromosome abnormalities after intracytoplasmic sperm injection. Lancet 346:1095. 7564797

[10] Sloter E, Nath J, Eskenazi B, Wyrobek AJ (2004) Effects of male age on the frequencies of germinal and heritable chromosomal abnormalities in humans and rodents. Fertil Steril 81:925–943. 15066442 10.1016/j.fertnstert.2003.07.043

[11] Nagaoka SI, Hassold TJ, Hunt PA (2012) Human aneuploidy: mechanisms and new insights into an age-old problem. Nat Rev Genet 13:493–504. 22705668 10.1038/nrg3245 PMC3551553

[12] Demaliaj E, Cerekja A, Piazze J (2012) Sex Chromosome Aneuploidies. In Storchova Z, ed. *Aneuploidy in Health and Disease.* Croatia; Press INTECH, 121–140.

[13] Sloter ED, Lowe X, Moore DHII, Nath J, Wyrobek AJ (2000) Multicolor FISH analysis of chromosomal breaks, duplications, deletions, and numerical abnormalities in the sperm of healthy men. Am J Hum Genet 67:862–872. 10961911 10.1086/303088 PMC1287891

[14] Bernardini L, Martini E, Geraedts JP, Hopman AH, Lanteri S, et al (1997) Comparison of gonosomal aneuploidy in spermatozoa of normal fertile men and those with severe male factor detected by in-situ hybridization. Mol Hum Reprod 3:431–438. 9239728 10.1093/molehr/3.5.431

[15] Pang MG, Hoegerman SF, Cuticchia AJ, Moon SY, Doncel GF, et al (1999) Detection of aneuploidy for chromosomes 4, 6, 7, 8, 9, 10, 11, 12, 13, 17, 18, 21, X and Y by fluorescence in-situ hybridization in spermatozoa from nine patients with oligoasthenoteratozoospermia undergoing intracytoplasmic sperm injection. Hum Reprod 14:1266–1273. 10325276 10.1093/humrep/14.5.1266

[16] Collodel G, Scapigliati G, Moretti E (2007) Spermatozoa and chronic treatment with finasteride: a TEM and FISH study. Arch Androl 53:229–233. 17852047 10.1080/01485010701426471

[17] Hook EB (1985) The impact of aneuploidy upon public health: mortality and morbidity associated with human chromosome abnormalities. Basic Life Sci 36:7–33. 4096707 10.1007/978-1-4613-2127-9_2

[18] Martin RH, Spriggs E, Ko E, Rademaker AW (1995) The relationship between paternal age, sex ratios, and aneuploidy frequencies in human sperm, as assessed by multicolor FISH. Am J Hum Genet 57:1395–1399. 8533769 PMC1801415

[19] McAuliffe ME, Williams PL, Korrick SA, Altshul LM, Perry MJ (2012) Environmental exposure to polychlorinated biphenyls and p, p′-DDE and sperm sex-chromosome disomy. Environ Health Perspect. 120:535–540. 22189045 10.1289/ehp.1104017 PMC3339457

[20] Robbins WA, Vine MF, Truong KY, Everson RB (1997a) Use of fluorescence in situ hybridization (FISH) to assess effects of smoking, caffeine, and alcohol on aneuploidy load in sperm of healthy men. Environ Mol Mutagen 30:175–183. 9329642 10.1002/(sici)1098-2280(1997)30:2<175::aid-em10>3.0.co;2-a

[21] Robbins WA, Meistrich ML, Moore D, Hagemeister FB, Weier HU, et al (1997b) Chemotherapy induces transient sex chromosomal and autosomal aneuploidy in human sperm. Nat Genet 16:74–78. 9140398 10.1038/ng0597-74

[22] Young-Ah You, El-Sayed A. Mohamed, Shin-Ae Oh, Myung-Geol Pang (2009) Optimized methods to maintain motility and viability in normozoospermic males. Kor Reprod Medicine 36:45–53.

[23] Pang MG, You YA, Park YJ, Oh SA, Kim DS, et al (2010) Numerical chromosome abnormalities are associated with sperm tail swelling patterns. Fertil Steril 94:1012–1020. 19505688 10.1016/j.fertnstert.2009.04.043

[24] Jeyenderan RS, Van der Ven HH, Perez-Pelaez M, Carbo BG, Zenevel LJD (1984) Development of an assay to assess the functional integrity of the human sperm membrane and its relationship to other semen characteristics. J Reperod Fertil 70:219–228. 10.1530/jrf.0.0700219 6694140

[25] Hossain AM, Osuamkpe CO, Nagamani M (2008) Extended culture of human spermatozoa in the laboratory may have practical value in the assisted reproductive procedures. Fertil Steril 89:237–239. 17482167 10.1016/j.fertnstert.2007.01.170

[26] Hossain A, Rizk B, Barik S, Thorneycroft I (1998) Time course of hypoosmotic swellings of human spermatozoa: evidence of ordered transition between swelling sub-types. Hum Reprod 13:1578–1583. 9688395 10.1093/humrep/13.6.1578

[27] Verheyen G, Joris H, Nagy Z, Steirteghem AV (1997) Comparison of different hypo-osmotic swelling solutions to select viable immotile spermatozoa for potential use in ICSI. Hum Reprod 3:195–203. 10.1093/humupd/3.3.195 9322097

[28] Martini AC, Molina RI, Tissera A, deCuneo MF (2006) Improving the predictive value of hypoosmotic swelling test in humans. Fertil Steril 85:1840–1842. 16677642 10.1016/j.fertnstert.2005.11.048

[29] Martin RH, Rademaker AW (1987) The effect of age, on the frequency of sperm chromosomal abnormalities in normal men. Am J Hum Genet 84:179–186. PMC1684194 3631081

[30] Mohamed EA, Pang MG (2012) Sperm aneuploidy and Male fertility. In Rossi S and Bianchi F ed. Aneuploidy, Etiology, Disorders and risk factors. NY; Press Nova Science publisher, Inc, 123–142.

[31] Tempaldo C, Vidal F, Estop A (2011) Aneuploidy in human spermatozoa. Cytogenet Genome Res 133:91–99. 21282942 10.1159/000323795

[32] Pfeffer J, Pang MG, Hoegerman SF, Osgood CJ, Stacey MW, et al (1999) Aneuploidy frequencies in semen fractions from ten oligoasthenoteratozoo-spermic patients donating sperm for intracytoplasmic sperm injection. Fertil Steril 72:472–478. 10519619 10.1016/s0015-0282(99)00279-4

[33] Vegetti W, Van Assche E, Frias A, Verheyen G, Bianchi MM, et al (2000) Correlation between semen parameters and sperm aneuploidy rates investigated by fluorescence in-situ hybridization in infertile men. Hum Reprod 15:351–365. 10655307 10.1093/humrep/15.2.351

[34] Templado C, Bosch M, Benet J (2005) Frequency and distribution of chromosome abnormalities in human spermatozoa. Cytogenet Genome Res 111, 199–205. 16192695 10.1159/000086890

[35] Johnson MD (1998) Genetic risks of intracytoplasmic sperm injection in the treatment of male infertility: recommendations for genetic counseling and screening. Fertil Steril 70:397–411. 9757865 10.1016/s0015-0282(98)00209-x

[36] Rodrigo L, Rubio C, Mateu E, Simón C, Remohí J, et al (2004) Analysis of chromosomal abnormalities in testicular and epididymal spermatozoa from azoospermic ICSI patients by fluorescence in-situ hybridization. Hum Reprod 19:118–123. 14688169 10.1093/humrep/deh012

[37] Meschede D, Louwen F, Eiben B, Horst J (1997) Intracytoplasmic sperm injection pregnancy with fetal trisomy 9p resulting from a balanced paternal translocation. Hum Reprod 12:1913–1914. 9363705 10.1093/humrep/12.9.1913

[38] Pehlivan T, Rubio C, Rodrigo L, Romero J, Remohi J, et al (2003) Impact of preimplantation genetic diagnosis on IVF outcome in implantation failure patients. Reprod Biomed Online 6:232–237. 12676006 10.1016/s1472-6483(10)61715-4

[39] Silber S, Escudero T, Lenahan K, Abdelhadi I, Kilani Z, et al (2003) Chromosomal abnormalities in embryos derived from testicular sperm extraction. Fertil Steril 79:30–38. 12524060 10.1016/s0015-0282(02)04407-2

[40] Jacobs PA, Hassold TJ (1995) The origin of numerical chromosome abnormalities. Adv Genet 33:101–133. 7484451 10.1016/s0065-2660(08)60332-6

